# Large triglyceride-rich lipoproteins in hypertriglyceridemia are associated with the severity of acute pancreatitis in experimental mice

**DOI:** 10.1038/s41419-019-1969-3

**Published:** 2019-09-30

**Authors:** Yue Zhang, Wenhua He, Cong He, Jianhua Wan, Xiao Lin, Xi Zheng, Lei Li, Xueyang Li, Xiaoyu Yang, Bingjun Yu, Xunde Xian, Yin Zhu, Yuhui Wang, George Liu, Nonghua Lu

**Affiliations:** 1 Department of Gastroenterology, The First Affiliated Hospital of Nanchang University, 330006 Nanchang, China; 2 Institute of Cardiovascular Sciences, Peking University Health Science Center, 100191 Beijing, China; 3 Key Laboratory of Molecular Cardiovascular Science, Ministry of Education, 100191 Beijing, China

**Keywords:** Dyslipidaemias, Dyslipidaemias, Acute pancreatitis, Acute pancreatitis

## Abstract

Hypertriglyceridemia severity is linked to acute pancreatitis prognosis, but it remains unknown why a portion of severe hypertriglyceridemia patients do not develop severe acute pancreatitis. To investigate whether hypertriglyceridemia subtypes affect acute pancreatitis progression, we analyzed two genetically modified hypertriglyceridemia mouse models—namely, glycosylphosphatidylinositol high-density lipoprotein binding protein 1 knockout (Gpihbp1−/−) and apolipoprotein C3 transgenic (ApoC3-tg) mice. Acute pancreatitis was induced by 10 intraperitoneal caerulein injections. Biochemical assays and pathological analysis were performed for the severity evaluation of acute pancreatitis. Plasma triglyceride-rich lipoproteins (TRLs), including chylomicrons and very low-density lipoprotein (VLDL), were collected via ultracentrifugation to evaluate their cytotoxic effects on primary pancreatic acinar cells (PACs). We found that the particle sizes of Gpihbp1−/− TRLs were larger than ApoC3-tg TRLs. Severe pancreatic injury with large areas of pancreatic necrosis in the entire lobule was induced in Gpihbp1−/− mice when plasma triglyceride levels were greater than 2000 mg/dL. However, ApoC3-tg mice with the same triglyceride levels did not develop large areas of pancreatic necrosis, even upon the administration of poloxamer 407 to further increase triglyceride levels. Meanwhile, in the acute pancreatitis model, free fatty acids (FFAs) in the pancreas of Gpihbp1−/− mice were greater than in ApoC3-tg mice. TRLs from Gpihbp1−/− mice released more FFAs and were more toxic to PACs than those from ApoC3-tg mice. Chylomicrons from patients showed the same effects on PACs as TRLs from Gpihbp1−/− mice. Gpihbp1−/− mice with triglyceride levels below 2000 mg/dL had milder pancreatic injury and less incidence of pancreatic necrosis than those with triglyceride levels above 2000 mg/dL, similar to Gpihbp1−/−mice with triglyceride levels above 2000 mg/dL but with fenofibrate administration. These findings demonstrated that hypertriglyceridemia subtypes with large TRL particles could affect acute pancreatitis progression and that chylomicrons showed more cytotoxicity than VLDL by releasing more FFAs.

## Introduction

Hypertriglyceridemia is the cause of acute pancreatitis, and it leads to a poor prognosis^1–5^. However, therapeutic guidelines for hypertriglyceridemia-associated acute pancreatitis are not well established. Triglyceride-lowering treatments have been implemented clinically, but their roles in improving the outcomes of acute pancreatitis remain unclear^6–10^. Therefore, fully understanding the mechanisms of acute pancreatitis under hypertriglyceridemia will be potentially valuable for clinical practice.

The pathogenesis of hypertriglyceridemia is complex and leads to multiple phenotypes enriched with various triglyceride-rich lipoproteins (TRLs) in plasma, including chylomicrons and/or very low-density lipoprotein (VLDL). Hypertriglyceridemia can be mainly divided into three subtypes, namely, Types I, IV, and V hyperlipidemia, according to the plasma TRL composition. It was reported that the subtypes were associated with acute pancreatitis episodes^11–14^ in the clinic, however, their relationship with prognosis and their underlying mechanisms are also worth exploring.

The hyperlipidemia subtypes are the result of interactions between genetic and environmental factors. Genetic mutations associated with lipoprotein lipase activity have been identified as the causes of severe hypertriglyceridemia. We chose two mouse hypertriglyceridemia models to mimic different hypertriglyceridemia subtypes in this study, including glycosylphosphatidylinositol-anchored high-density lipoprotein binding protein 1 knockout (Gpihbp1−/−) and apolipoprotein C3 transgenic (ApoC3-tg) mice. Gpihbp1 is necessary for TRLs to bind to the vascular endothelium during triglyceride hydrolysis by lipoprotein lipase^15,16^. Gpihbp1 deficiency is one contributing factor to Type I hyperlipidemia with hereditary recurrent acute pancreatitis^17–19^. ApoC3 is involved in the inhibition of TRL hydrolysis and the clearance of their remnants^20^. In some studies performed on human subjects, elevated plasma ApoC3 levels were related to insulin resistance accompanied by hypertriglyceridemia^21–23^. Plasma from the two kinds of mice showed a milky appearance; in Gpihbp1−/− mice, this plasma was mainly enriched with chylomicrons^15,16,24^, whereas in ApoC3-tg mice, it was mainly enriched with VLDL and TRL remnants^25,26^.

Havel, et al^27^. initially proposed the theory that excessive fatty acids beyond albumin binding can damage the vascular endothelium and pancreatic acinar cells (PACs). Fatty acids in the pancreas are not only derived from lipoproteins but also from peripancreatic adipose tissue, which caused cytotoxicity to PACs mainly due to unsaturated fatty acids but not saturated fatty acids^28,29^ through a variety of mechanisms^30–35^. FFAs are generally considered as an important link between hypertriglyceridemia and acute pancreatitis.

To identify the relationship of hypertriglyceridemia subtypes and acute pancreatitis progression and reveal the underlying mechanisms, we used two kinds of hypertriglyceridemia mouse models with caerulein induction to evaluate the severity of acute pancreatitis, tested the injury of PACs treated with different kinds of TRLs, and examined FFA production and their classifications. We hope our results can explain how both triglyceride levels and the types of TRLs can affect the death of pancreatic acinar cells and thus the severity of acute pancreatitis. These discoveries will help clinicians to develop more precise diagnostic and therapeutic strategies for high-risk patients.

## Results

### Two hypertriglyceridemia mouse models presented different TRL sizes in plasma

**Fig. 1:**
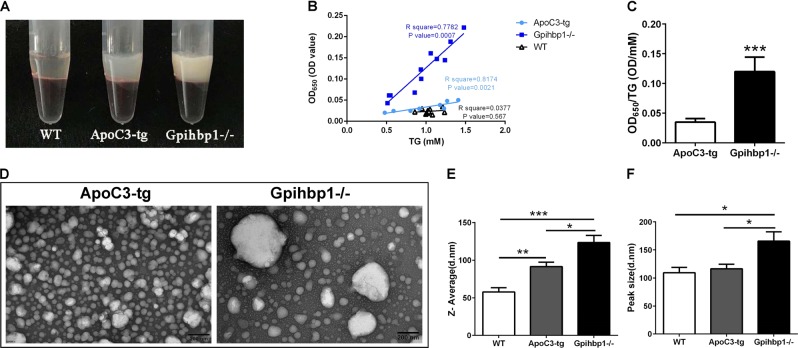
Different size of triglyceride-rich lipoprotein particles between Gpihbp1−/− and ApoC3-tg mice. **a** The graph showing plasma appearance of ApoC3-tg and Gpihbp1−/− mice with the consistent triglyceride concentration of 3471.25 mg/dL and 3498.59 mg/dL, and clear appearance of plasma of wild-type mouse as a negative control. **b** Correlation between triglyceride level and OD value of plasma solution of ApoC3-tg and Gpihbp1−/− mice. **c** The ratio of OD value and triglyceride level between ApoC3-tg and Gpihbp1−/− mice. Each value was the mean ± SD, 8–10 samples per group. **d** Transmission electron microscopy of triglyceride-rich lipoprotein particles from ApoC3-tg and Gpihbp1−/− mice, in which each sample was diluted to consistent triglyceride levels (40 mg/dL). Scale bar = 200 nm, ×30,000. **e** The representative graphs of particles distribution of plasma from wild-type, ApoC3-tg and Gpihbp1−/− mice, in which each plasma sample was diluted to consistent triglyceride levels (40 mg/dL). **e**, **f** Comparison of Z-Average size and peak size of plasma lipoprotein particles between wild-type, ApoC3-tg and Gpihbp1−/− mice, in which each plasma sample was diluted to consistent triglyceride levels (40 mg/dL). Each value was the mean ± SEM, 5–8 samples per group. *p < 0.05 or **p < 0.01 or ***p < 0.001. OD optical density, TG triglyceride, WT wild-type

Because the two hypertriglyceridemia mouse models, namely, Gpihbp1−/− and ApoC3-tg mice, are generated by different genetic modifications, their TRLs may be different when representing the two types of hypertriglyceridemia. We analyzed the characteristics of the TRLs in the plasma from the two models. We found that plasma from Gpihbp1−/− mice had a more milky-white appearance than that from ApoC3-tg mice, although they displayed the same triglyceride levels (Fig. 1a). The optical density was positively associated with the triglyceride concentration in the plasma from both hypertriglyceridemia mice models, while the turbidity (shown as OD_650_/triglyceride) of the Gpihbp1−/− mice plasma was significantly higher than the ApoC3-tg mice plasma (Fig. 1b, c). These results suggested that Gpihbp1−/− TRLs may be larger than ApoC3-tg TRLs. For further evidence, we examined their TRLs with transmission electron microscopy, and the results showed much larger TRL particles from Gpihbp1−/− mice than ApoC3-tg mice at the same triglyceride concentration (Fig. 1d); however, there were fewer particles in the Gpihbp1−/− samples than in the ApoC3-tg samples. Additionally, using a Malvern Zetasizer Nano, average and peak sizes of the plasma lipoprotein particles in Gpihbp1−/− mice were identified as significantly larger than those in ApoC3-tg mice (Fig. 1e). Their representative distribution is shown in Supplementary Fig. 1. This evidence suggested that large chylomicron particles are the main TRLs in Gpihbp1−/− mice, while small VLDL particles are the main TRLs in ApoC3-tg mice plasma. Therefore, Gpihbp1−/− and ApoC3-tg mice are obviously two different types of hypertriglyceridemia models with different TRL types.

### Large areas of pancreatic necrosis were induced in Gpihbp1−/− mice but not in ApoC3-tg mice by 10 caerulein injections when triglyceride levels were greater than 2000 mg/dL

**Fig. 2:**
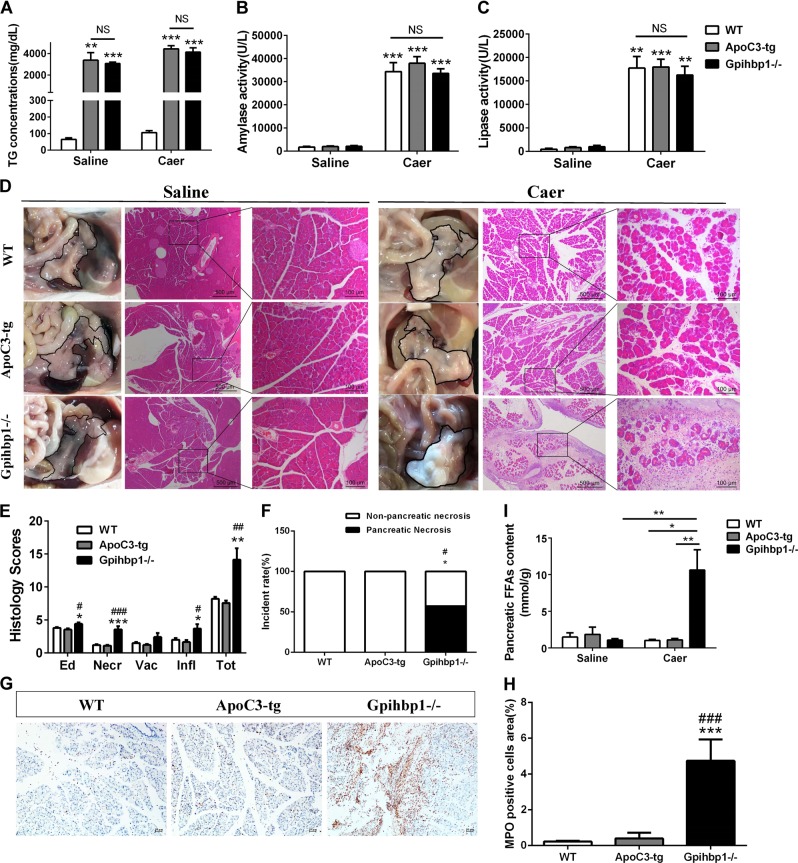
The comparison of pancreatic injury, inflammation and free fatty acids of pancreas between wild-type, ApoC3-tg and Gpihbp1−/− mice in caerulein-induced acute pancreatitis. **a** Triglyceride concentration of plasma between wild-type, ApoC3-tg and Gpihbp1−/− mice before caerulein or normal saline treatment. **b**, **c** Plasma amylase and lipase activity at 12th hour after the first injection of caerulein between these three groups. Each value was the mean ± SEM for *n* = 7–10 mice in acute pancreatitis groups administrated 10 intraperitoneal injections of caerulein (50 μg/kg) and *n* = 3–4 in saline groups administrated equal volume normal saline. **d** Appearance and representative photomicrographs of H&E-stained section of pancreas of wild-type, ApoC3-tg and Gpihbp1−/− mice in acute pancreatitis groups and normal saline groups. Appearance of pancreas of each sample was delineated with black line. **e** Pathological scores of the pancreas of wild-type, ApoC3-tg and Gpihbp1−/− mice in acute pancreatitis groups. Scale bar = 500 or 100 μm. Each value was the mean ± SEM for *n* = 7–10. **f** Incidence rates of pancreatic necrosis of wild-type, ApoC3-tg and Gpihbp1−/− mice in acute pancreatitis groups. *n* = 7–10. **g** Immunohistochemistry evaluation of myeloperoxidase in pancreas of wild-type, ApoC3-tg and Gpihbp1−/− mice in acute pancreatitis groups. Scale bar = 10 μm. **h** Semiquantitative results of the area ratio of myeloperoxidase positive cells among wild-type, ApoC3-tg and Gpihbp1−/− mice in acute pancreatitis groups. Each value was the mean ± SEM, *n* = 5. **i** Free fatty acids concentration of pancreas of these three groups in acute pancreatitis groups and normal saline groups. Each value was the mean ± SEM for *n* = 5 in acute pancreatitis groups and *n* = 3–5 in normal saline groups. *p < 0.05 or **p < 0.01 or ***p < 0.001 vs wild-type group. ^#^*p* < 0.05 or ^##^*p* < 0.01 or ^###^*p* < 0.001 vs ApoC3-tg group. WT wild-type, TG triglyceride, NS no significance, Caer caerulein, Ed edema, Necr, necrosis, Vac vacuolisation, Infl inflammation, FFAs free fatty acids

We previously found large individual differences in the triglyceride levels in the Gpihbp1−/− and ApoC3-tg mouse models (Supplementary Fig. 2). We chose mice with high triglyceride levels (>2000 mg/dL) from both models for the induction of acute pancreatitis. There was no significant difference in the triglyceride levels between the Gpihbp1−/− and ApoC3-tg mice (Fig. 2a). However, interestingly, the Gpihbp1−/− group showed albescent saponification-like foci in the pancreas (Fig. 2d). Pancreatic cross-sections stained with hematoxylin and eosin (H&E) showed an exciting result in the Gpihbp1−/− group, with large patchy necrotic areas (Fig. 2d and Supplementary Fig. 3) involving pancreatic acini and the surrounding tissues. This was much different from the scattered acinar cell necrosis in the caerulein-induced ApoC3-tg mice and other previous reports on caerulein-induced models^3,31,36^. We defined the pathological features of this patchy necrosis of the whole lobule as pancreatic necrosis compared to the patchy areas of the pancreas without enhancement displayed by contrast-enhanced computed tomography in patients. Magnetic renounce imaging also showed that the Gpihbp1−/− mouse had many liquid-signal areas, which indicated severe pancreatic injury (Supplementary Fig. 4). Sirius red staining revealed the presence of greater collagen amounts in the necrosis areas (Supplementary Fig. 3). We found that large areas of pancreatic necrosis were absent from both the wild-type and ApoC3-tg groups (Fig. 2f).

The results also showed that amylase and lipase activities were dramatically higher in the caerulein-treatment groups than in the normal saline groups; however, there were no significant differences among the wild-type, ApoC3-tg and Gpihbp1−/− mice in the caerulein-treatment groups (Fig. 2b, c). Because plasma amylase and lipase activities were found to peak at 12 h after the first caerulein injection in wild-type mice in the pilot study (Supplementary Fig. 5), plasma amylase and lipase activity tests were performed at this time.

Morphologically, compared to the normal saline groups, pancreatic swelling in the caerulein-induced groups was visible from the gross appearance (Fig. 2d). By scoring the H&E-stained sections, we found that edema, inflammatory infiltration, and pancreatic acinar cell necrosis were more severe in the Gpihbp1−/− group than in the wild-type and ApoC3-tg groups (Fig. 2e). Myeloperoxidase staining showed more neutrophil infiltration in pancreatic tissues from the Gpihbp1−/− group compared to the wild-type and ApoC3-tg groups (Fig. 2g, h), suggesting stronger acute inflammatory response in Gpihbp1−/− group.

When acute pancreatitis caused TRL exposure to pancreatic lipase in pancreatic tissues, triglycerides would be hydrolyzed to release FFAs, resulting in acinar cell damage^27,28,34,37^. Via lipid extraction, we found that FFAs in the pancreas were significantly higher in Gpihbp1−/− than in ApoC3-tg and wild-type mice after caerulein induction, and there were no differences among the three control groups (Fig. 2i). This finding indicated that TRLs in Gpihbp1−/− mice might release large amounts of FFAs locally to further damage the pancreas after acute pancreatitis induction.

### ApoC3-tg mice did not develop large areas of pancreatic necrosis even when plasma triglyceride levels increased to levels greater than in Gpihbp1−/− mice

**Fig. 3:**
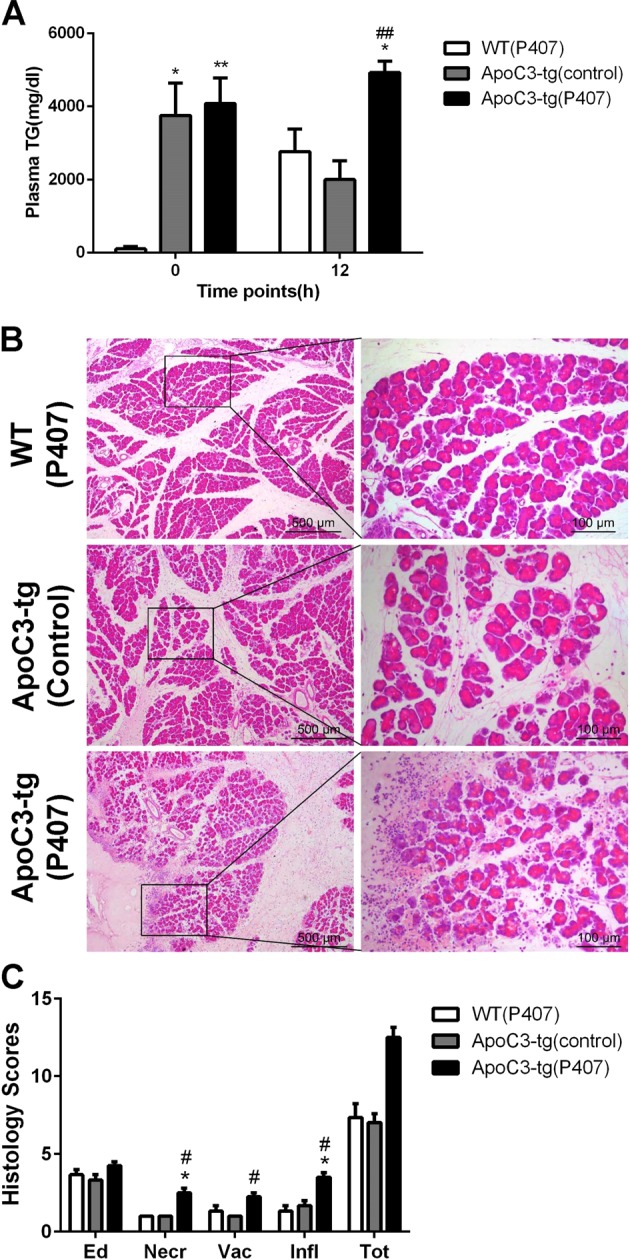
The comparison of pancreatic injury between ApoC3-tg mice with and without the administration of Poloxamer 407. **a** Plasma triglyceride levels before acute pancreatitis induction and 12 h after the first injection of caerulein between wild-type mice with Poloxamer 407 administration, ApoC3-tg without Poloxamer 407 and ApoC3-tg with Poloxamer 407. **b** Representative photomicrographs of H&E-stained section of pancreas of these three groups after acute pancreatitis induction. **c** pathological scores of the pancreas between these three groups after acute pancreatitis induction. Scale bar = 500 or 100 μm. *p < 0.05 or **p < 0.01 or ***p < 0.001 vs wild-type mice with Poloxamer 407 administration. ^#^*p* < 0.05 or ^##^*p* < 0.01 or ^###^*p* < 0.001 vs ApoC3-tg without poloxamer 407. Values are means ± SD of 3–4 animals per group. WT wild-type, TG triglyceride, Ed edema, Necr necrosis, Vac vacuolisation, Infl inflammation, P407 Poloxamer 407

We found that the plasma triglyceride concentration in ApoC3-tg mice decreased by 50%, but not in Gpihbp1−/− mice 12 h after caerulein injection (Supplementary Fig. 6). Therefore, decreased triglyceride levels may lead to less injury without large areas of pancreatic necrosis. We used poloxamer 407 to inhibit the reduction in plasma triglyceride levels during acute pancreatitis^36,38^. The results showed that poloxamer 407 treatment increased plasma triglyceride levels after caerulein injection in ApoC3-tg mice (Fig. 3a) and aggravated pancreatic injury, but large areas of pancreatic necrosis were not induced (Fig. 3b, c). We concluded that the large areas of pancreatic necrosis did not occur regardless of how high the triglyceride levels of small particle TRLs were. Therefore, our results suggested that the particle size, rather than the plasma triglyceride levels, is a key factor for severe pancreatic damage.

### TRLs from Gpihbp1−/− mice were more toxic to primary pancreatic acinar cells

**Fig. 4:**
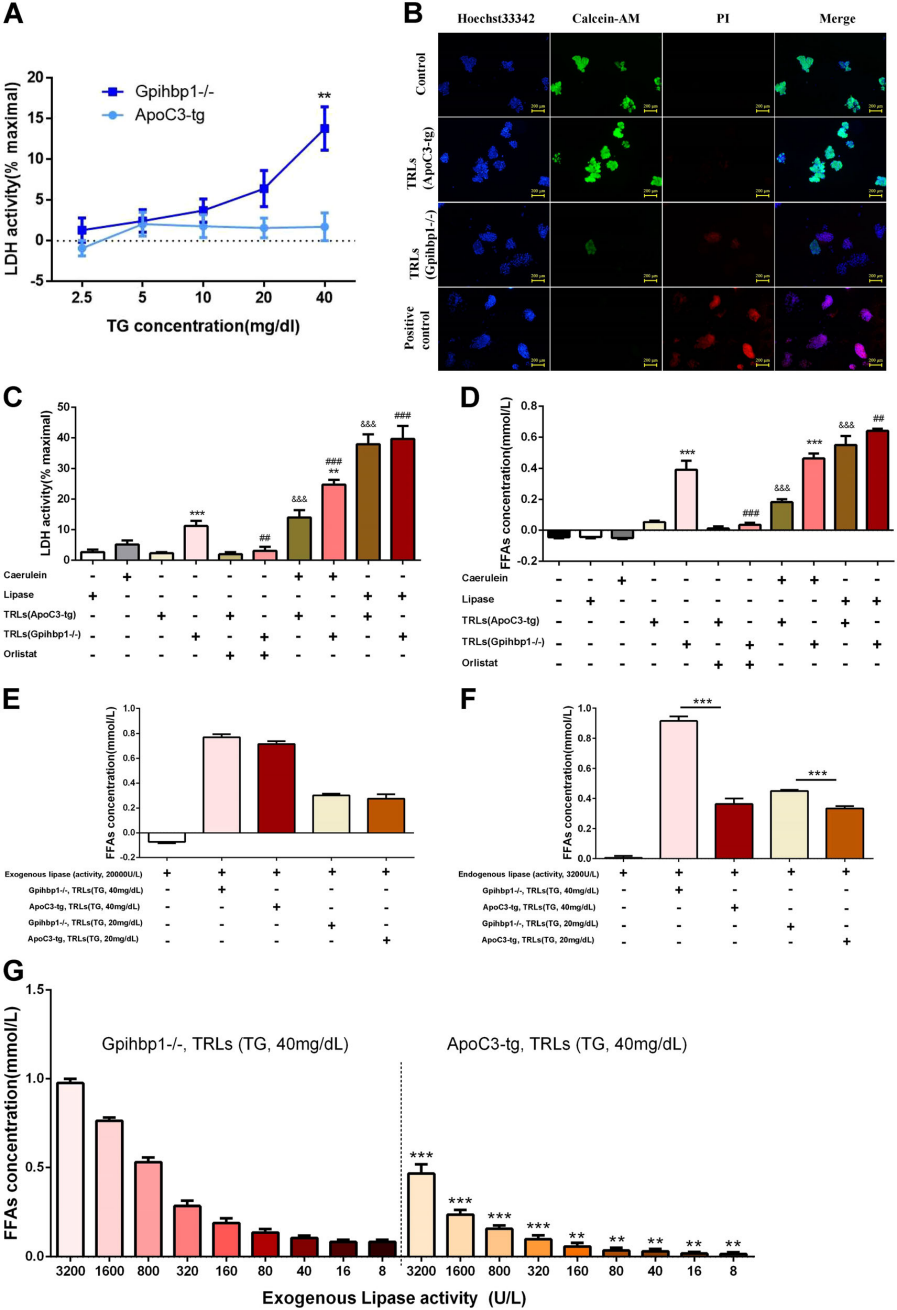
The comparison of cytotoxicity to primary pancreatic acinar cells between triglyceride-rich lipoproteins from Gpihbp1−/− and ApoC3-tg mice. **a** Cell necrosis was quantified as % lactate dehydrogenase leakage after primary pancreatic acinar cells being incubated for 30 min with triglyceride-rich lipoproteins from Gpihbp1−/− and ApoC3-tg mice when triglyceride concentrations of TRLs were 2.5, 5, 10, 20, 40 mg/dL, respectively. *p < 0.05 or **p < 0.01 or ***p < 0.001 vs ApoC3-tg group. Three samples for each group for two independent experiments. **b** Representative graphs of Hoechst33342, PI and calcein-AM fluorescence staining of primary pancreatic acinar cells after incubated with triglyceride-rich lipoproteins from ApoC3-tg and Gpihbp1−/− mice with triglyceride concentration of 40 mg/dL for 30 min. Scale bar = 200 μm. **c** Percent Lactate dehydrogenase leakage, **d** Levels of free fatty acids when primary pancreatic acinar cells were incubated with caerulein, lipase, triglyceride-rich lipoproteins of ApoC3-tg and Gpihbp1−/− mice with triglyceride concentration of 40 mg/dL alone or accompanied with caerulein or orlistat or lipase for 30 min. Scale bar = 200 μm. Three samples for each group for two independent experiments. *p < 0.05 or **p < 0.01 or ***p < 0.001 TRLs (Gpihbp1−/−) group vs TRLs (ApoC3-tg) group or TRLs (Gpihbp1−/−) + caerulein group vs TRLs(ApoC3-tg) + caerulein group. ^#^*p* < 0.05 or ^##^*p* < 0.01 or ^###^*p* < 0.001 vs TRLs (Gpihbp1−/−) group. ^&^*p* < 0^.^05 or ^&&^*p* < 0.01 or ^&&&^*p* < 0.001 vs TRLs (ApoC3-tg) group. **e** The release of free fatty acids when triglyceride-rich lipoproteins (triglyceride concentration, 40 mg/dL or 20 mg/dL) from ApoC3-tg and Gpihbp1−/− mice was incubated with pig pancreatic lipase (lipase activity, about 20,000 U/L) for 30 min. **f** The release of free fatty acids when triglyceride-rich lipoproteins (triglyceride concentration, 40 mg/dL or 20 mg/dL) from ApoC3-tg and Gpihbp1−/− mice was incubated with supernatant (lipase activity, about 3000 U/L) collected through primary pancreatic acinar cells incubated with caerulein (100 nM) for 30 mins. **g** The release of free fatty acids when triglyceride-rich lipoproteins from ApoC3-tg and Gpihbp1−/− mice with triglyceride level of 40 mg/dL was incubated with various activity of pig pancreatic lipase for 30 min. *p < 0.05 or **p < 0.01 or ***p < 0.001 vs Gpihbp1−/− with corresponding lipase activity. Three samples for each group for two independent experiments. PI propidium iodide, FFAs free fatty acids, TRLs triglyceride-rich lipoproteins, LDH lactate dehydrogenase

To investigate why mice with large but not small TRL particles can result in severe pancreatic necrosis, we examined the effects of different TRLs on primary pancreatic acinar cells (PACs). First, we found that PACs released lactate dehydrogenase (LDH) in a concentration-dependent manner when they were incubated with Gpihbp1−/− TRLs, but not when incubated with ApoC3-tg TRLs, and the LDH concentration was significantly higher with Gpihbp1−/− TRL treatment than ApoC3-tg TRL treatment when the triglyceride concentration was 40 mg/dL (Fig. 4a). Among these PACs, there was more propidium iodide (PI) uptake by cells and fewer calcein-AM-stained cells after treatment with Gpihbp1−/− TRLs than with ApoC3-tg TRLs. (Fig. 4b). Hence, it has been suggested that Gpihbp1−/− TRLs might damage pancreatic acinar cells more than ApoC3-tg TRLs.

Many previous studies have proven that FFAs hydrolyzed from triglycerides were cytotoxic^28,31,39^. In this study, we found that both LDH (activity) and FFAs (concentration) increased more when PACs were incubated with Gpihbp1−/− TRLs than ApoC3-tg TRLs at a TG concentration of 40 mg/dL (Fig. 4c, d). However, the pancreatic lipase inhibitor orlistat could inhibit the increase in both LDH and FFAs (Fig. 4c, d). When PACs were stimulated with caerulein, both LDH and FFAs increased after incubation with Gpihbp1−/− and ApoC3-tg TRLs, though Gpihbp1−/− TRLs still released more LDH and FFAs than ApoC3-tg TRLs (Fig. 4c, d). Trypan Blue staining showed consistent results on PACs damage (Supplementary Fig. 7). Furthermore, when treated with an excess of lipase (20,000 U/L), FFA production and LDH release increased to the same level indicated that PACs were equally damaged when incubated with Gpihbp1−/− or ApoC3-tg TRLs (Fig. 4c, d). This finding suggested that different size TRLs have different hydrolysis efficiencies for pancreatic lipase. Gpihbp1−/− TRLs was more liable to releasing FFAs to cause more severe PACs injury than ApoC3-tg TRLs under pathophysiological levels of lipase.

To additionally identify the production of FFAs from different TRLs, we incubated TRLs with lipase (commercial or supernatant from caerulein-stimulated PACs) without PACs. As shown in Fig. 4e, when the lipase activity was up to 20,000 U/L, there were no significant differences in the production of FFAs between TRLs from the Gpihbp1−/− and ApoC3-tg mice at triglyceride levels of 40 or 20 mg/dL. However, this excess of pancreatic lipase would not present in vivo, regardless of the physiological or pathological state.

However, when TRLs from Gpihbp1−/− or ApoC3-tg mice were incubated with supernatants from caerulein-stimulated PACs (lipase activity at 3565 ± 223 U/L), more FFAs were released from Gpihbp1−/− TRLs than ApoC3-tg TRLs (Fig. 4f). Furthermore, we incubated TRLs from Gpihbp1−/− or ApoC3-tg mice with lipase (commercial, gradient activity from 8 to 3200 U/L), and we found that more FFAs were released from Gpihbp1−/− TRLs than ApoC3-tg TRLs (Fig. 4g). Thus, these results indicated that large TRL particles released more FFAs under in vivo lipase activity.

Moreover, to identify whether the FFA compositions of the two types of TRLs were different, which is generally considered as a characteristic of TRLs from different sources, we analyzed the fatty acids compositions of the two TRL types by liquid chromatography mass spectrometry (LC-MS). Higher concentrations of eicosapentaenoic acid (C20:5), docosahexaenoic acid (C22:6,and linoleic acid (C18:2) were found in Gpihbp1−/− TRLs compared to ApoC3-tg TRLs (Supplementary Fig. 8b), which displayed consistent total FFA concentrations (data not shown).

### Human chylomicrons were more toxic to primary pancreatic acinar cells than human VLDL

**Fig. 5:**
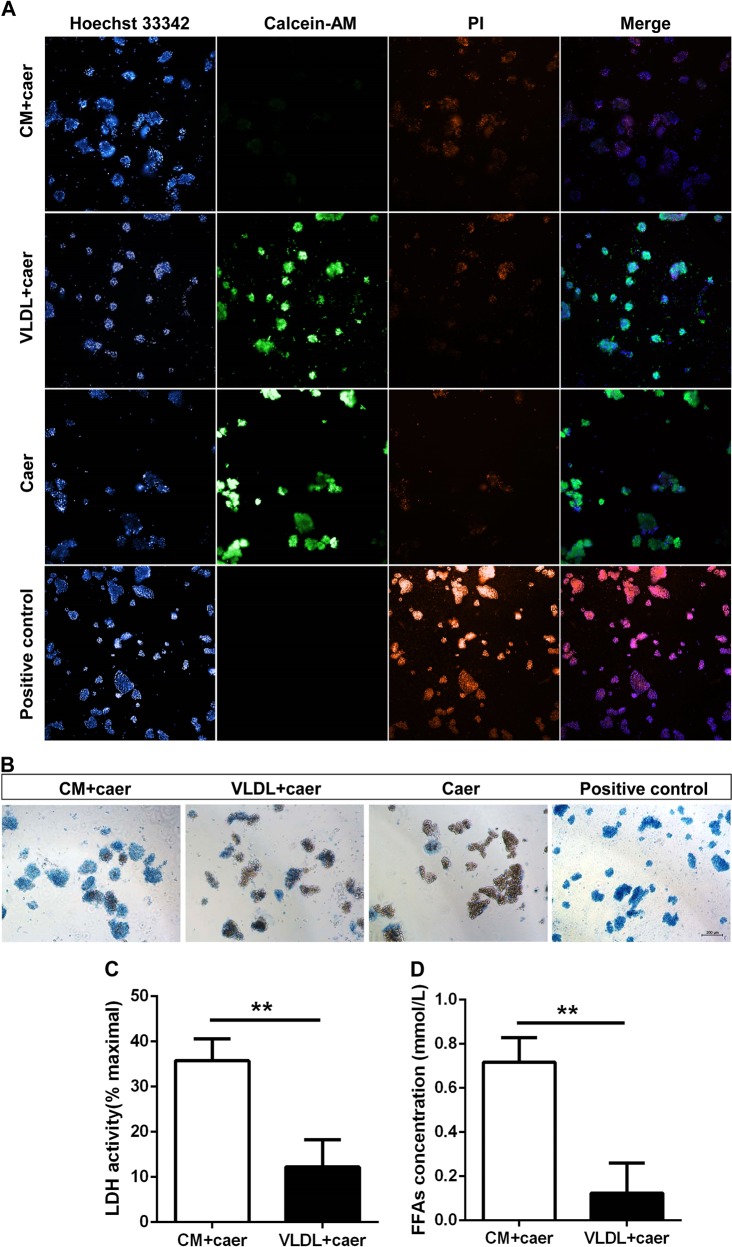
The comparison of cytotoxicity to primary pancreatic acinar cells between chylomicron and very low-density lipoprotein from acute pancreatitis patients with hypertriglyceridemia. **a** Representative graphs of Hoechst33342, PI and calcein-AM fluorescence staining of primary pancreatic acinar cells after incubated with caerulein (100 nM) combining chylomicron or very low-density lipoprotein with triglyceride concentration of 40 mg/dL for 30 min. Photographed by high-throughput cell imaging system, Scale bar = 100 μm. **b** Representative graphs of Trypan Blue Dye of primary pancreatic acinar cells when they were incubated with caerulein (100 nM) combining chylomicron or very low-density lipoprotein with triglyceride concentration of 40 mg/dL for 30 min. Scale bar = 200 μm. **c**, **d** Lactate dehydrogenase leakage % and free fatty acids concentration when primary pancreatic acinar cells were incubated with caerulein (100 nM) combining chylomicron or very low-density lipoprotein with triglyceride concentration of 40 mg/dL for 30 min. *p < 0.05 or **p < 0.01 or ***p < 0.001. Three samples for each group. PI propidium iodide, CM chylomicron, VLDL very low-density lipoprotein, LDH lactate dehydrogenase, FFAs free fatty acids

After identifying that large TRL particles had stronger cytotoxicity to PACs than small TRLs using mouse blood samples, we further investigated whether human TRLs have the same effects as murine TRLs. We separated chylomicrons and VLDL from acute pancreatitis patients with hypertriglyceridemia. PI, Trypan blue and calcein-AM staining showed that there were more necrotic PACs after incubation with chylomicrons than VLDL (Fig. 5a, b). Like mouse TRLs, the LDH and FFAs in culture medium with human chylomicrons increased to greater than that with VLDL. (Fig. 5c, d).

### Lowering plasma triglycerides in Gpihbp1−/− mice with large TRL particles significantly reduced pancreatic necrosis

**Fig. 6:**
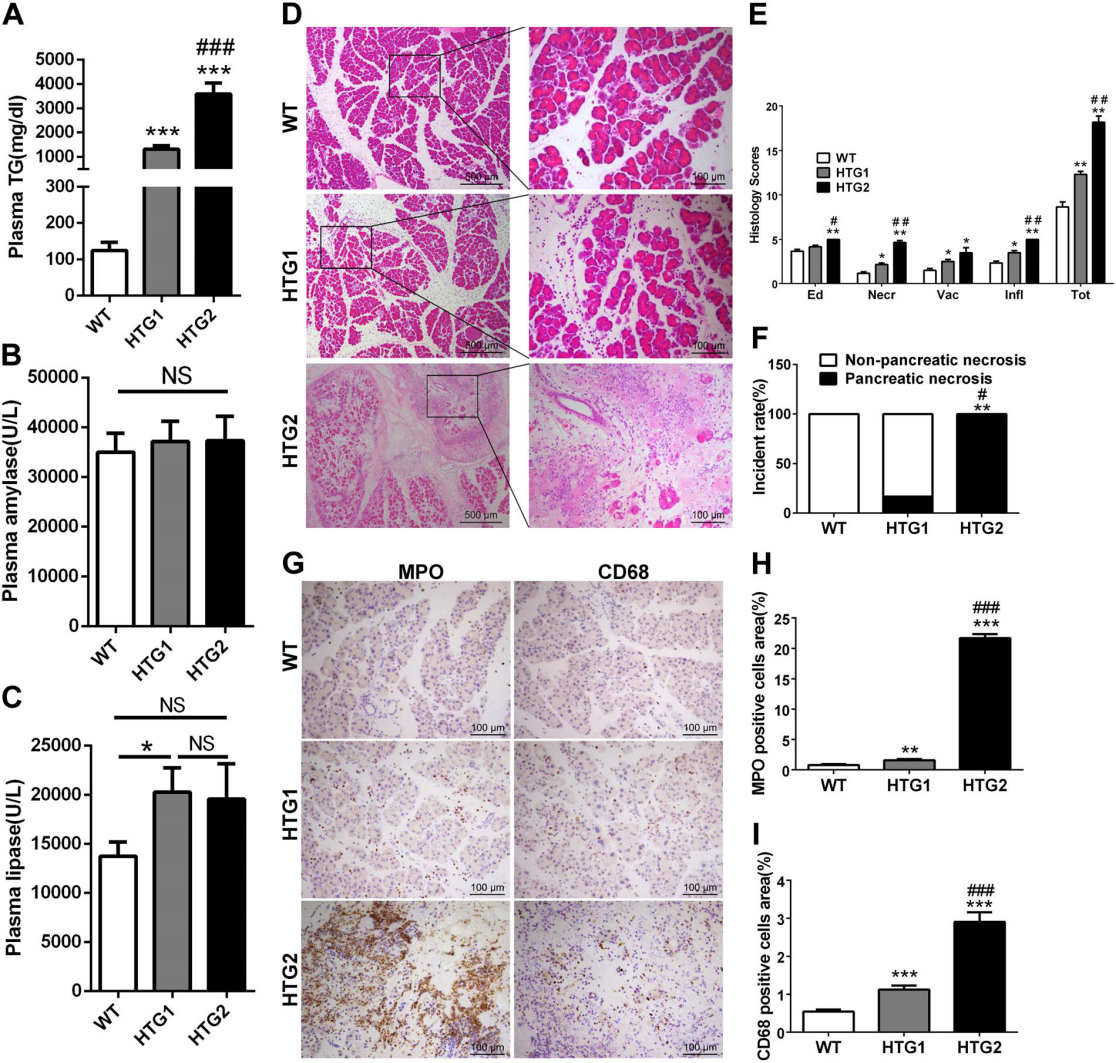
The comparison of pancreatic injury and inflammation between Gpihbp1−/− mice with different HTG severity in caerulein-induced acute pancreatitis. **a** Triglyceride concentration of plasma before caerulein treatment between wild-type, HTG1 (triglyceride concentration, 1000–2000 mg/dL) and HTG2 mice(triglyceride concentration, å 2000 mg/dL). **b**, **c** Plasma amylase and lipase activity at 12^th^ hour after the first injection of caerulein (50 μg/kg, 10 times, hourly) between these three groups. **d** Representative photomicrographs of H&E-stained section of pancreas in wild-type, HTG1 and HTG2 mice after acute pancreatitis induction. Scale bar = 500 or 100 μm. **e** Pathological scores of the pancreas in wild-type, HTG1 and HTG2 mice after acute pancreatitis induction. **f** Incidence rates of pancreatic necrosis between these three groups after acute pancreatitis induction. Each value was the mean ± SEM for *n* = 6. **g** Immunohistochemistry evaluation for myeloperoxidase and CD68 in pancreas between wild-type, HTG1 and HTG2 mice after acute pancreatitis induction. Scale bar = 100 μm. **i** Semiquantitative results of the area ratio of myeloperoxidase and CD68-positive cells among wild-type, HTG1 and HTG2 mice after acute pancreatitis induction. Each value was the mean ± SEM for *n* = 3–5. *p < 0.05 or **p < 0.01 or ***p < 0.001 vs wild-type group. ^#^*p* < 0.05 or ^##^*p* < 0.01 or ^###^*p* < 0.001 vs HTG1 group. WT wild-type, TG triglyceride, Ed edema, Necr necrosis, Vac vacuolisation, Infl inflammation, FFAs free fatty acids, MPO myeloperoxidase

As described in the Supplementary Fig. 2, because of the individual differences in Gpihbp1−/− mice triglyceride levels, we chose individuals with triglyceride levels greater than 2000 mg/dL for the experiments above. Here, to investigate the relationship of triglyceride levels and the incidences of large areas of pancreatic necrosis in Gpihbp1−/− mice with large TRL particle sizes, we compared low triglyceride level Gpihbp1−/− mice (<2000 mg/dL, HTG1) with high triglyceride level mice (>2000 mg/dL, HTG2) on the severity of acute pancreatitis (Fig. 6a). After caerulein induction, both amylase and lipase activities were not significantly different among the wild-type, HTG1 and HTG2 groups (Fig. 6b, c). Morphologically, the HTG1 group showed more severe pancreatic injury than the wild-type mice but considerably less pancreatic damage than the HTG2 mice (Fig. 6d, e). The incidence rate of pancreatic necrosis was 100% in the HTG2 group, while the incidence rate was 0% in the wild-type group and 17% in the HTG1 group (Fig. 6f). More myeloperoxidase and CD68-positive cells were found in pancreatic tissues from the HTG2 group than the wild-type and HTG1 groups (Fig. 6g–i). Interleukin 1β (IL-1β) and monocyte chemotactic protein 1 (MCP-1) mRNA expression in the pancreas was significantly higher in the HTG2 group than in the HTG1 and wild-type groups; mRNA expression of vascular cell adhesion molecule-1 (VCAM-1) was higher in the HTG1 group than in wild-type group (Supplementary Fig. 9).

**Fig. 7:**
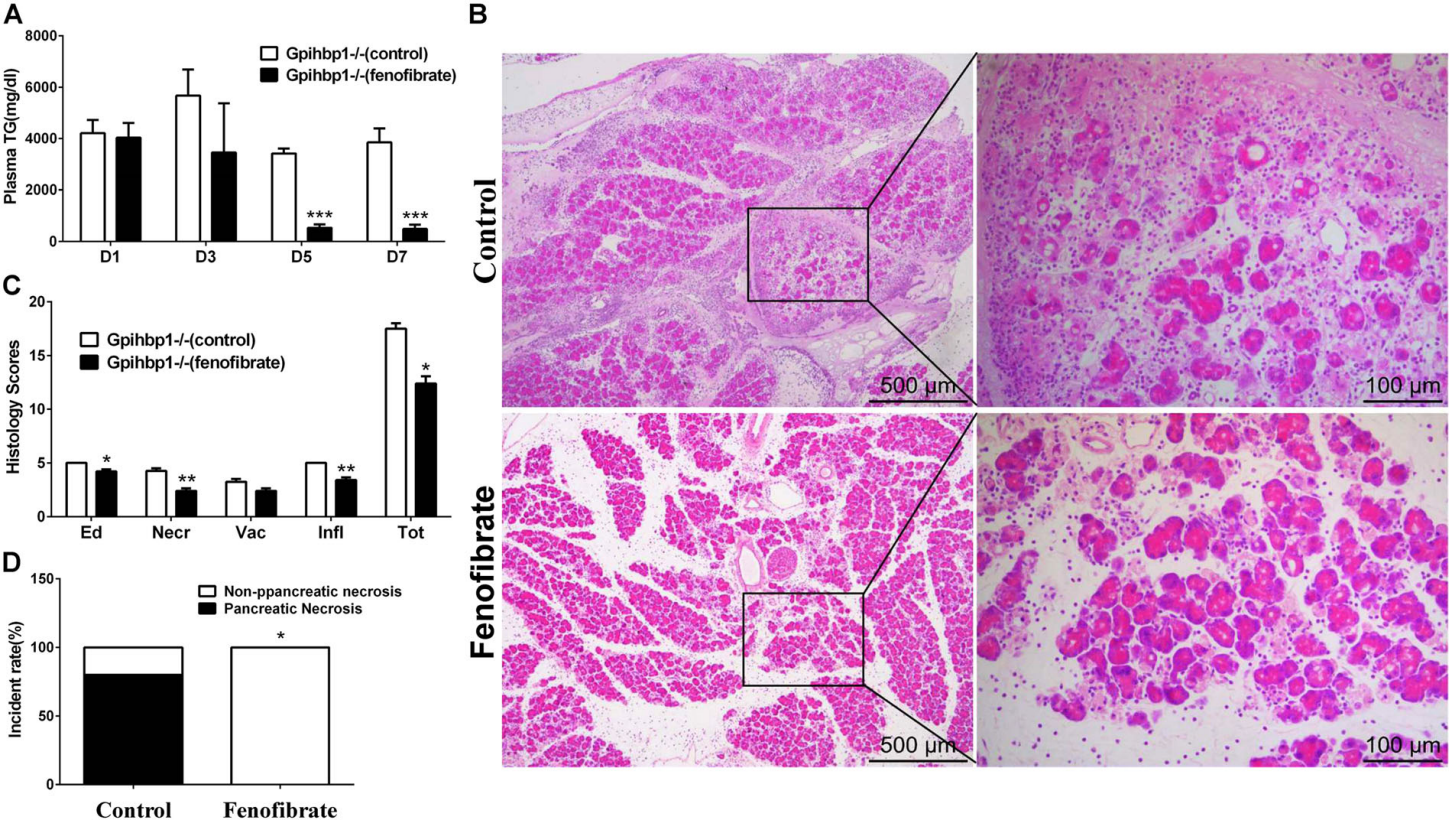
The comparison of pancreatic injury and the incidence of pancreatic necrosis between Gpihbp1−/− mice with or without fenofibrate treatment in caerulein-induced acute pancreatitis. **a** Plasma triglyceride levels in day1 (before fenofibrate treatment), day 3, 5, and 7 (after fenofibrate treatment) in Gpihbp1−/−(control) and Gpihbp1−/−(fenofibrate) mice. **b** Representative photomicrographs of H&E-stained section of pancreas after acute pancreatitis induction (caerulein, 50 μg/kg, 10 times, hourly) in Gpihbp1−/−(control) and Gpihbp1−/−(fenofibrate) mice. **c** Pathological scores of the pancreas after acute pancreatitis induction in Gpihbp1−/− (control) and Gpihbp1−/− (fenofibrate) mice. Scale bar = 500 or 100 μm. **d** Incidence rates of pancreatic necrosis after acute pancreatitis induction in Gpihbp1−/− (control) and Gpihbp1−/− (fenofibrate) mice. *p < 0.05 or **p < 0.01 or ***p < 0.001 vs Gpihbp1−/−(control) (*n* = 5–6). Each value was the means ± SD in histograms. D1 the day before fenofibrate was given, D3, D5, and D7 the third, fifth, and seventh day after fenofibrate was given. TG triglyceride, Ed edema, Necr necrosis, Vac vacuolisation, Infl inflammation

We lowered the plasma triglyceride concentration by fenofibrate administration to investigate the preventive effects on acute pancreatitis exacerbation in Gpihbp1−/− mice. We found that plasma triglyceride concentrations were dramatically lowered in Gpihbp1−/− mice after 6 days of fenofibrate administration before acute pancreatitis was induced (Fig. 7a). On the seventh day, when triglyceride levels were stable at low levels (487.4 ± 353.3 mg/dL) due to fenofibrate treatment, we induced acute pancreatitis by caerulein injection. We found that Gpihbp1−/− mice with fenofibrate treatment showed a reduced incidence of pancreatic necrosis and injury by H&E staining (Fig. 7b–d).

## Discussion

It is well known that patients with type I hyperchylomicronemia exhibit extremely higher acute pancreatitis episodes than patients with types IV (hyperpre-β-lipoproteinemia) and V (mixed type hyperpre-β-lipoproteinemia and chylomicronemia) hyperlipidemia^13^. It has also been reported that among Chinese patients, variants of lipoprotein lipase or its regulating genes, which have been linked with hyperchylomicronemia, increased the risk of acute pancreatitis^40^. Therefore, clinical practices have revealed that chylomicrons have a close relationship with acute pancreatitis.

In this study, two kinds of hypertriglyceridemia mouse models allowed us to compare the effects of different kinds of TRLs on the exacerbation of acute pancreatitis, and the large areas of pancreatic necrosis induced by 10 caerulein injections in Gpihbp1−/− mice allowed us to examine the special effects of large TRL particles almost at a glance. Gpihbp1−/− mice showed very high plasma triglyceride levels because of the almost complete deficiency in lipoprotein lipase function, resulting in chylomicronemia^17^. ApoC3 overexpression results in increased VLDL and remnants by inhibiting lipoprotein lipase activity and remnants clearance and promoting VLDL synthesis^20^. Different genetic modifications can lead to different TRL metabolism disorders and the presence of larger TRL particles in Gpihbp1−/− than those in ApoC3-tg. Our results showed that even with the same high triglyceride levels, pancreatic injury exacerbation and the presence of large areas of pancreatic necrosis only occurred in Gpihbp1−/− mice, not in ApoC3-tg mice. Moreover, poloxamer 407 treatment in ApoC3-tg mice further increased triglyceride levels, but did not to induce large areas of pancreatic necrosis in this type of hypertriglyceridemia. This phenomenon is very similar to the inconsistent prognosis of acute pancreatitis in patients even though they have the same high triglyceride levels. Additionally, we were excited to find that the very severe necrosis associated with large patchy pancreatic parenchymal tissues mimicking patients could be induced in Gpihbp1−/− mice. We considered that animal models and patients with hypertriglyceridemia may have the same mechanisms for the aggravation of acute pancreatitis. Our results showed that the hypertriglyceridemia subtype is a key factor during this process due to the different types of TRLs damaging PACs differently.

At present, some studies have shown that FFAs, hydrolysis products of TRLs, play a key role in hypertriglyceridemia-related pancreatic injury^28,30^. In our study, more FFAs were extracted from the pancreas of Gpihbp1−/− mice than ApoC3-tg mice after caerulein induction, suggesting that greater FFA production may be the reason for occurrence of pancreatic necrosis in Gpihbp1−/− mice. In in vitro experiments, more FFAs were released and more cytotoxicity was directed towards PACs from Gpihbp1−/− TRLs than ApoC3-tg TRLs, which was consistent with quantities of FFAs produced in vivo. This further confirmed that these pathological processes were important during the progression of hypertriglyceridemia-associated acute pancreatitis. Our results also showed that TRLs were the substrates of pancreatic lipase. Additionally, under an in vivo environment, the activity of pathologically released pancreatic lipase can promote more hydrolysis of large TRLs particles (chylomicrons). Taken together, these results suggested that large TRL particles may release more FFAs than small TRL particles in response to pancreatic lipase.

However, future research should examine the manners by which pancreatic lipase affects TRLs, such as its enzymatic kinetics and coproteins or other auxiliaries. Although ApoC3 can inhibit lipoprotein lipase activity, it remains unclear whether ApoC3 overexpression also inhibit pancreatic lipase activity, resulting in less FFA release. Moreover, whether other lipoprotein lipase activity suppressor genes can also influence pancreatic lipase activity also needs to by confirm.

The increased FFAs hydrolyzed from large TRL particles locally in pancreatic tissue might be the reason for the increased severe pancreas injury until the presence of pancreatic necrosis through a vicious circle in Gpihbp1−/− mice due to large TRL particles. Chylomicrons separated from patient plasma released more FFAs and showed stronger cytotoxic effects on PACs than VLDL, suggesting to some extent that this mechanism also occurs in humans.

In addition, we also found more unsaturated fatty acids in TRLs from Gpihbp1−/− than from ApoC3-tg mice (Supplementary Fig. 8b). Unsaturated fatty acids are dietary-derived, essential fatty acids for mammals^41,42^. This result also explained that TRLs in Gpihbp1−/− mice were mainly enriched in chylomicrons of intestinal origin. Moreover, it has been reported that unsaturated fatty acids rather than saturated fatty acids caused more damage to PACs^28,39^. Therefore, more unsaturated fatty acids in TRLs may be another reason for the exacerbation and occurrence of pancreatic necrosis in acute pancreatitis.

Orlistat was effective at inhibiting pancreatic lipase activity in vitro, in animal experiments, triglyceride-lowering treatment significantly reduced the severity of acute pancreatitis and pancreatic necrosis. In clinical practice, necrotizing pancreatitis involve pancreatic parenchymal necrosis, extra-pancreatic necrosis or their combination, whereby patients with pancreatic parenchymal necrosis alone or in combination more often suffered from systemic and local complications^43–50^. Due to the lack of guidance for the treatment of acute pancreatitis with hypertriglyceridemia, the National Association for the Improvement of Blood (ASFA) only classifies these patients as a Class III indication for plasma exchange, which can be used individually^51^. Our results suggested that hypertriglyceridemia-associated acute pancreatitis prognosis should refer to the hypertriglyceridemia subtype and particle size, and severe acute pancreatitis caused by extensive pancreatic necrosis should be prevented by lowering triglyceride levels which potentially decrease large TRL particles. This study suggested that patients with elevated chylomicrons would be more likely to benefit from emergency lipid-lowering therapy, which requires the performance of future clinical studies to confirm. Additionally, our findings also propose a potential value for the development of methods to remove chylomicrons but not VLDL.

The study also suggested that inhibition of FFA production may be an effective way to inhibit pancreatic acinar cell injury. However, orlistat, a lipase inhibitor, fails to enter the pancreatic vascular bed and extracellular space of pancreatic acinar cells through blood circulation due to its insolubility.

In summary, a severe necrotic pancreatitis mouse model with hypertriglyceridemia by caerulein induction was established in this study. In future, many studies on mechanisms or preventive treatments for acute pancreatitis with hypertriglyceridemia could be performed on this animal model. We here by this model, we found that both triglyceride levels and the TRL composition are the key factors for the development of acute pancreatitis, especially pancreatic necrosis. Triglycerides derived from large TRL particles, such as chylomicrons, rather than small TRL particles, might be more harmful to the pancreas after the onset of acute pancreatitis.

## Materials and methods

### Animal experiments

In this study, Gpihbp1−/− mice with a C57BL/6 background were obtained from MMRRC Mice Services (strain name: B6;129S5-Gpihbp1tm1Lex/Mmucd; stock no. 032334-UCD) and human ApoCIII transgenic mice with a C57BL/6 background were obtained from JAX Mice Services (strain name: B6; CBA-Tg [APOC3]3707Bres/J; stock no. 006907) through a Chinese agent (Vital River Laboratories, Beijing, China) at the Institute of Cardiovascular Sciences of Peking University (Beijing, China). Because we had previously found that CD1 mice were more sensitive to caerulein induction^52^, in this study, both Gpihbp1−/− and ApoC3-tg mice with a C57BL/6 background were crossbred with CD1 background mice for seven generations to generate their CD1 lines for our experiments.

Genotyping of Gpihbp1−/− and ApoC3-tg mice was performed by PCR analysis of genomic DNA extracted from their tails^53^. Female and male mice aged 10–15 weeks and weighting ~32–38 g were used in this study. Animals were maintained under a 12-h light/12-h dark cycle at 24 °C with standard laboratory chow and water ad libitum. The Principles of Laboratory Animal Care (NIH publication no. 85Y23, revised 1996) were upheld during the study. All experiments were performed in accordance with protocols approved by the Animal Care Committee, Peking University Health Science Center (LA2015012). Experiments were also approved by the Institutional Animal Care and Use Committee of Nanchang University and followed the rules set forth in the Guide for the Care and Use of Laboratory Animals.

For evaluating how the hypertriglyceridemia subtypes affected the progress of acute pancreatitis, mice were divided into experimental groups for acute pancreatitis induction and control groups for normal saline treatment, with both consisting of the following three groups: wild-type group (normal plasma triglyceride levels), ApoC3-tg (hypertriglyceridemia model), and Gpihbp1−/− (hypertriglyceridemia model) groups. The two kinds of hypertriglyceridemia mice with similar triglyceride levels (greater than 2000 mg/dL) were selected for the induction of acute pancreatitis to compare their pancreatic injuries.

Poloxamer 407 is a hydrophilic triblock copolymer comprised of polyoxyethylene and polyoxypropylene units and has been reported to induce hypertriglyceridemia with few side effects^54^. Poloxamer 407 can increase serum triglyceride concentrations up to 2000 mg/dL by directly inhibiting the activity of both lipoprotein lipase and hepatic lipase^55–57^. A previous study showed that poloxamer 407 could be used long-term in wild-type mice to establish hypertriglyceridemia mice for research on acute pancreatitis^36^. In our study, we used poloxamer 407 once to transiently increase triglyceride levels in ApoC3-tg mice during acute pancreatitis induction. Furthermore, to identify whether increasing triglyceride levels in ApoC3-tg mice during acute pancreatitis induction could exacerbate pancreatic injury, ApoC3-tg mice with triglyceride levels greater than 2000 mg/dL after poloxamer 407 treatment were compared with ApoC3-tg mice without poloxamer 407 after acute pancreatitis induction. The wild-type mice treated with poloxamer 407 in this experiment were designed to exclude the effects of poloxamer 407 administration on pancreatic injury compared to further increasing plasma triglycerides during acute pancreatitis induction in ApoC3-tg mice.

To understand how triglyceride levels in the Gpihbp1−/− mouse model affected the progression of acute pancreatitis, Gpihbp1−/− mice were divided into the HTG1 group (Gpihbp1−/− mice with triglyceride levels greater than 1000 mg/dL and less than 2000 mg/dL) and HTG2 group (Gpihbp1−/− mice with triglyceride levels greater than 2000 mg/dL). Wild-type mice with normal triglyceride levels were considered as controls. Then, acute pancreatitis was induced in these groups. Moreover, for further evidence, Gpihbp1−/− mice with triglyceride levels greater than 2000 mg/dL were treated with fenofibrate until their plasma triglycerides decreased to a stable low level as well as solvent as a control. Then, acute pancreatitis was induced in the two groups to verify whether dramatically decreasing plasma triglyceride levels in Gpihbp1−/− mice with triglyceride levels greater than 2000 mg/dL could prevent pancreatic injury.

### Induction of acute pancreatitis and sample collection

Because patients are usually attacked by acute pancreatitis after eating, in this study, all mice were fed chow diets and water ad libitum before the induction of acute pancreatitis. A sterile solution of caerulein (AnaSpec, Inc., Fremont, USA) was prepared in normal saline at a concentration of 10 µg/ml and administered intraperitoneally to induce acute pancreatitis by 10 hourly injections at 50 µg/kg body weight. Equivalent normal saline was injected as a non-acute pancreatitis control.

Poloxamer 407 (Sigma) was intraperitoneally injected into ApoC3-tg mice at 200 mg/kg; the two control groups were ApoC3-tg mice without poloxamer 407 treatment and wild-type mice with poloxamer 407 treatment (200 mg/kg). Poloxamer 407 was administered half an hour after the first caerulein injection.

Fenofibrate (Recipharm Fournier, France) in 0.5% carboxymethyl cellulose sodium (CMC-Na) at 100 mg/kg body weight was administered by intragastric infusion three times per day for several days. Caerulein-induced acute pancreatitis was promoted until plasma triglycerides decreased to a stable low level in the fenofibrate treatment group.

Blood samples were collected from the orbital vein with heparin sodium for anticoagulation at 0, 12, and 24 h after the first caerulein injection. Plasma was isolated by centrifugation at 4000 rpm for 10 min at 4 °C. Mice were sacrificed by overdose with pentobarbital (Fort Dodge Animal Health, Fort Dodge, Iowa, USA) at 24 h after the first caerulein injection. Part of the pancreas was fixed in 4% buffered paraformaldehyde for paraffin sectioning and the other part was frozen in −80 °C for mRNA and lipid extraction.

### Biochemistry assay

In this study, plasma triglyceride levels were measured by the enzymatic terminal method using commercial kits (Biosino Biotechnology and Science Inc, Beijing, China). The FFA concentrations were also examined by commercial kits (Wako LabAssay, Japan) as described in their manuals. Amylase and lipase activities were measured by the Catalyst Dx Chemistry Analyzer (IDEXX Laboratories, Inc., Westbrook, ME) using the DRI-CHEM method. Plasma samples for amylase and lipase tests were preprocessed by ultracentrifugation at 25,000 rpm for 30 min at 4 °C to avoid lipemia interference. Amylase and lipase activities were tested from plasma collected at 12 h after the first caerulein injection using the premise that their activities in mice plasma reached a peak in the 12 h after the first caerulein injection (Supplementary Fig. 5). The lactic dehydrogenase (LDH) activity of cell culture medium was measured using a commercial kit via a 30 min reaction at room temperature in the dark (Dojindo, Japan).

### Histopathological assay

Paraffin-embedded pancreatic tissue was cut into 4-μm sections for H&E and Sirius Red staining by usual methods. Two investigators were blinded to evaluate the severity of edema, inflammation, necrosis, and vacuolization according to previously described standards in the H&E staining image analysis^58,59^, in which the evaluation of necrosis was modified to sensitively discriminate the degree of necrosis (Supplementary Table 1). The incidence rate of pancreatic necrosis was calculated by the ratio of the number of mice with large areas of necrosis to the total number of mice within the same treatment group. Neutrophil and macrophage infiltration were evaluated by immunohistochemical staining using anti-myeloperoxidase rabbit polyclonal antibody (Abcam, ab9535) and anti-CD68 rabbit polyclonal antibody (Boster, BA3638), respectively. Briefly, paraffin-embedded sections were deparaffinized and incubated with 0.3% H_2_O_2_ solution to remove endogenous peroxidase activity. Antigen retrieval was performed with citrate buffer (pH 6.8). Then, goat serum was used to block nonspecific antigens. The sections were incubated with diluted (1:200) anti- myeloperoxidase polyclonal antibody or diluted (1:400) anti-CD68 polyclonal antibody. The secondary antibody was a goat anti-rabbit secondary antibody. A diaminobenzene horseradish peroxidase color development kit was used for stain development. The sections were counterstained with hematoxylin and dehydrated. The myeloperoxidase areas and CD68-positive cells were calculated using the ImageJ software.

### Quantitative PCR assay

Total RNA from pancreatic tissues was extracted with the Trigol Reagent (Dingguo Biotechnology Limited Company, Beijing, China). Gel electrophoresis was performed to confirm no degradation after reverse transcription with Super Script II RT (Invitrogen, Carlsbad, Calif). Inflammatory cytokine expression was quantified by quantitative PCR with EVA Green (Invitrogen, Carlsbad, Calif). The expression of the tested genes was normalized to glyceraldehyde-phosphate dehydrogenase (GAPDH) expression. The primer sequences are as follows:

IL-1β,

forward-GCCACCTTTTGACAGTGATGAG and

reverse-AAGGTCCACGGGAAAGACAC;

CCCL2/MCP1,

forward-CCACAACCACCTCAAGCACT and

reverse-TAAGGCATCACAGTCCGAGTC;

VCAM-1,

forward-AGTTGGGGATTCGGTTGTTCT and

reverse-CCCCTCATTCCTTACCACCC; and

GAPDH,

forward-TGATGACATCAAGAAGGTGGTGAAG and

reverse-TCCTTGGAGGCCATGTAGGCCAT

### Isolation of total triglyceride-rich lipoproteins, chylomicrons, and very low-density lipoprotein

TRLs include chylomicrons and VLDL. The two kinds of TRLs have different Svedberg flotation units due to their different triglyceride contents, with chylomicrons and VLDL > 400 and 20–400 Svedberg flotation units, respectively^60^. TRLs were isolated by density gradient ultracentrifugation according to Redgrave and Carlson^61^ with slightly modification by Karpe et al^62^.

Plasma was collected from ApoC3-tg and Gpihbp1−/− mice and then was stored at 4 °C for further isolation within 72 h. Their total TRLs were isolated in normal saline (density 1.006 g/ml solution) by density gradient centrifugation (Beckman Coulter, OptimaTML-100K) at 230,000 × *g* for 18 h. The white floating stratum was carefully collected and stored at 4 °C for further in vitro experiments.

Plasma was collected from three acute pancreatitis patients with hypertriglyceridemia using sodium citrate anticoagulant tubes within 48 h after the onset of acute pancreatitis and then stored at 4 °C for further isolation. Chylomicrons were isolated from plasma in normal saline by density gradient centrifugation (Beckman Coulter, OptimaTML-100K) at 100,000 × *g* for 20 min. The white stratum containing the chylomicrons was carefully collected. The remaining liquid, which was also in normal saline, was separated by density gradient centrifugation (Beckman Coulter, OptimaTML-100K) at 230,000 × *g* for an additional 18 h. The white stratum containing the VLDL was carefully collected. The chylomicrons and VLDL were stored at 4 °C until further in vitro experiments.

### Assessment of plasma lipoprotein particle sizes

Isolated TRL particles diluted by normal saline to a triglyceride concentration of 40 mg/dL were stained by uranyl acetate and then analyzed by transmission electron microscopy (JEM-1400PLUS, JEOL Ltd.) with ×30000 magnification. All plasma samples diluted by normal saline to a triglyceride concentration of 40 mg/dL was also measured by dynamic light scattering using a Malvern Zetasizer Nano ZS90 (Malvern Instruments, Worcestershire, UK) to determine the particle average and peak sizes, which works based on the Mie theory. Besides, the plasma was collected from mice on a normal diet. All sizes measurements were carried out at 25 °C. Then the average and peak sizes of lipoproteins were analyzed, in which the analyzed peak size of lipoproteins of wild-type mice came from the highest peak for wild-type mice presented several peaks of lipoproteins.

### Lipids extraction from tissues

We extracted lipids from tissues using Folch’s method^63^. For the measurement of lipids in pancreatic tissue, we performed body circulatory perfusion with phosphate buffer saline (0.01 M) before the pancreas was removed. The lipids in the pancreas were extracted by homogenizing with 2:1 chloroform-methanol (v/v), and the lipids in the organic phase were dried with nitrogen. The lipids were dissolved with 3% Triton X-100 before measurement.

### Lipid extraction from the hydrolysis solution containing triglyceride-rich lipoproteins and LC-MS detection of free fatty acids

TRLs from ApoC3-tg and Gpihbp1−/− mice with final triglyceride concentrations at 200 mg/dL were hydrolyzed with excess pancreatic enzyme (20000 U/L) for 30 min at 37 °C to release FFAs. FFA concentrations in hydrolysis solution were not significantly different between the Gpihbp1−/− and ApoC3-tg mice (data not shown). Lipids were extracted according to a modified method by Bligh and Dyer^37,64^ with 400 μl of chloroform: methanol (2:1) per 100 μl of hydrolysis solution. The mixture was vortexed and centrifuged at 836 × *g* for 30 min at 4 °C. The organic solvent was evaporated with a nitrogen stream. Then, the pellets were successively dissolved in 20 μl of methanol:chloroform (1:1) and 60 μl of isopropanol:acetonitrile:water (2:1:1). An LC-20AD Shimadazu pump system and API 5500Q-TRAP mass spectrometer (AB SCIEX, Framingham, MA) were used for the separation and analysis. Mobile phase A was prepared with 10 mM ammonium acetate in 60% acetonitrile with 0.5% formic acid. Mobile phase B was prepared with 10 mM ammonium acetate in isopropanol:acetonitrile (9:1) with 0.1% formic acid. The multiple reaction monitoring (MRM) list of metabolites is shown in the Supplementary Table 2. A representative chromatograph of separation of multiple fatty acids by LC/MS from the Gpihbp1−/− and ApoC3-tg mice is shown in Supplementary Fig. 8a.

### Primary pancreatic acinar cell isolation and cell culture

Primary pancreatic acinar cells (PACs) were prepared based on two previous studies^65,66^ from adult ICR (CD1) mice. We used 1000 U of collagenase purchased from Worthington (LS005273) for PAC isolation. The cells were cultured in DMEM/F-12 with HEPES (Gibco, Germany) at 37 °C with 5% CO_2_ and used for experiments within 4 h after isolation. Greater than 95% cell viability was confirmed by trypan blue staining. Then the isolated cells were used for further in vitro experiments.

PACs were incubated with TRLs from ApoC3-tg and Gpihbp1−/− mice at different triglyceride concentrations (2.5, 5, 10, 20, and 40 mg/dL) for 30 min. To assess PAC necrosis and the LDH activity of the cell supernatant, Trypan Blue and fluorescent staining of PACs were performed as described above and below. Orlistat (1 mM, Aladdin, USA) was used to inhibit the activity of pancreatic lipase and caerulein (100 nM) to stimulate pancreatic lipase secretion in the cell culture experiments. FFAs were also measured as described as above.

PACs were incubated with chylomicrons or VLDL at the same triglyceride concentrations (40 mg/dL) from plasma of acute pancreatitis patients with hypertriglyceridemia in combination with caerulein (100 nM). To assess PAC necrosis and the LDH activity of the cell supernatant, Trypan Blue and fluorescent staining of PACs was performed. FFAs were also measured.

### Pancreatic acinar cells staining

Propidium iodide (PI, Dojindo, Japan), calcein-acetoxymethyl (calcein-AM, Dojindo) and Hoechst 33342 (Dojindo) were incubated with the cells at 37 °C and 5% CO_2_ for 15 min at a final concentration of 1, 1.5, and 3.6 μM, respectively, and then were washed once for Inverted Fluorescence Microscope analysis or photographed by a high-throughput cell imaging system at the following fluorescent wavelengths: PI, λex = 530 nm and λem = 580 nm; Calcein-AM, λex = 490 nm and λem = 515 nm; and Hoechst33342, λex = 350 nm and λem = 460 nm. Trypan Blue (Sigma, USA) staining was performed at a final concentration of 0.01% in cell medium and then observed under optical light using an inverted microscope.

### Statistical analysis

Results of animal and cell experiments were presented as mean ± SD or mean ± SEM. Data were analyzed with one-way ANOVA analysis of variance by Bonferroni test or by independent sample *t*-test after undergoing a normal distribution test. Histological scores were analyzed by Mann–Whitney rank-sum test. Analysis of incidence of patchy necrosis was performed with Chi-squared test. *P* < 0.05 was considered statistically significant. All data were processed with SPSS software.

## Supplementary information

Supplementary Table 1

Supplementary Table 2

Supplementary Figures
