# Supplementary material for: Large triglyceride-rich lipoproteins in hypertriglyceridemia are associated with the severity of acute pancreatitis in experimental mice
Source: Cell Death Dis. 2019 Sep 30;10(10):728. doi: 10.1038/s41419-019-1969-3 (PMC6768872; doi:10.1038/s41419-019-1969-3)
Supplement: Supplementary file 1 — Supplementary Table 1 [file 41419_2019_1969_MOESM1_ESM.docx]

**Suppl Table 1. The standards for quantitative estimation of the morphological degrees of pancreatic injury**

| **Score** | **1** | **2** | **3** | **4** | **5** |
| --- | --- | --- | --- | --- | --- |
| **Edema** | Local interlobular swelling | Global lobular swelling | Swelling of acinar spaces | Swelling of intercellular spaces | Island-like acini or cells |
| **Necrosis** | ＜2% | 2%-10% | 10%-20% | 20%-40% | ＞40% |
| **Vacuolization** | ＜1% | 1%-5% | 5%-10% | 10%-20% | ＞20% |
| **Inflammatory cell infiltration** | ＜5 Cells | 5-10 Cells | 10-20 Cells | 20-30 Cells | ＞30 Cells |

The extent of pancreatic parenchymal necrosis and vacuolation were assessed as the percentage of pancreatic acinar cell necrosis and vacuolation. Inflammatory cell infiltration was calculated by calculating the number of inflammatory cells by 400×magnification, and the average of 10 visual fields needed to be evaluated.
