# Supplementary material for: Large triglyceride-rich lipoproteins in hypertriglyceridemia are associated with the severity of acute pancreatitis in experimental mice
Source: Cell Death Dis. 2019 Sep 30;10(10):728. doi: 10.1038/s41419-019-1969-3 (PMC6768872; doi:10.1038/s41419-019-1969-3)
Supplement: Supplementary file 2 — Supplementary Table 2 [file 41419_2019_1969_MOESM2_ESM.docx]

**Suppl Table 2.** Metabolite MRM list for targeted LC-MS detection

| Metabolites | Q1 Mass (Da) | Q3 Mass (Da) |
| --- | --- | --- |
|  |  |  |
| FFA 16:0 | 301.2 | 255.2 |
| FFA 18:1 | 327.3 | 281.2 |
| FFA 14:0 | 273.2 | 227.2 |
| FFA 18:0 | 329.3 | 283.3 |
| FFA 18:2 | 325.2 | 325.2 |
| FFA 20:0 | 311.3 | 311.3 |
| FFA 22:0 | 339.3 | 339.3 |
| FFA 20:5 | 301.2 | 301.2 |
| FFA 20:2 | 307.3 | 307.3 |
| FFA 20:4 | 349.2 | 349.2 |
| FFA 22:6 | 327.2 | 327.2 |
| FFA 20:1 | 309.3 | 309.3 |
| FFA 24:0 | 367.4 | 367.4 |
| FFA 26:0 | 395.4 | 395.4 |
