# Supplementary material for: Large triglyceride-rich lipoproteins in hypertriglyceridemia are associated with the severity of acute pancreatitis in experimental mice
Source: Cell Death Dis. 2019 Sep 30;10(10):728. doi: 10.1038/s41419-019-1969-3 (PMC6768872; doi:10.1038/s41419-019-1969-3)
Supplement: Supplementary file 3 — Supplementary Figures [file 41419_2019_1969_MOESM3_ESM.docx]

**Supplementary information**


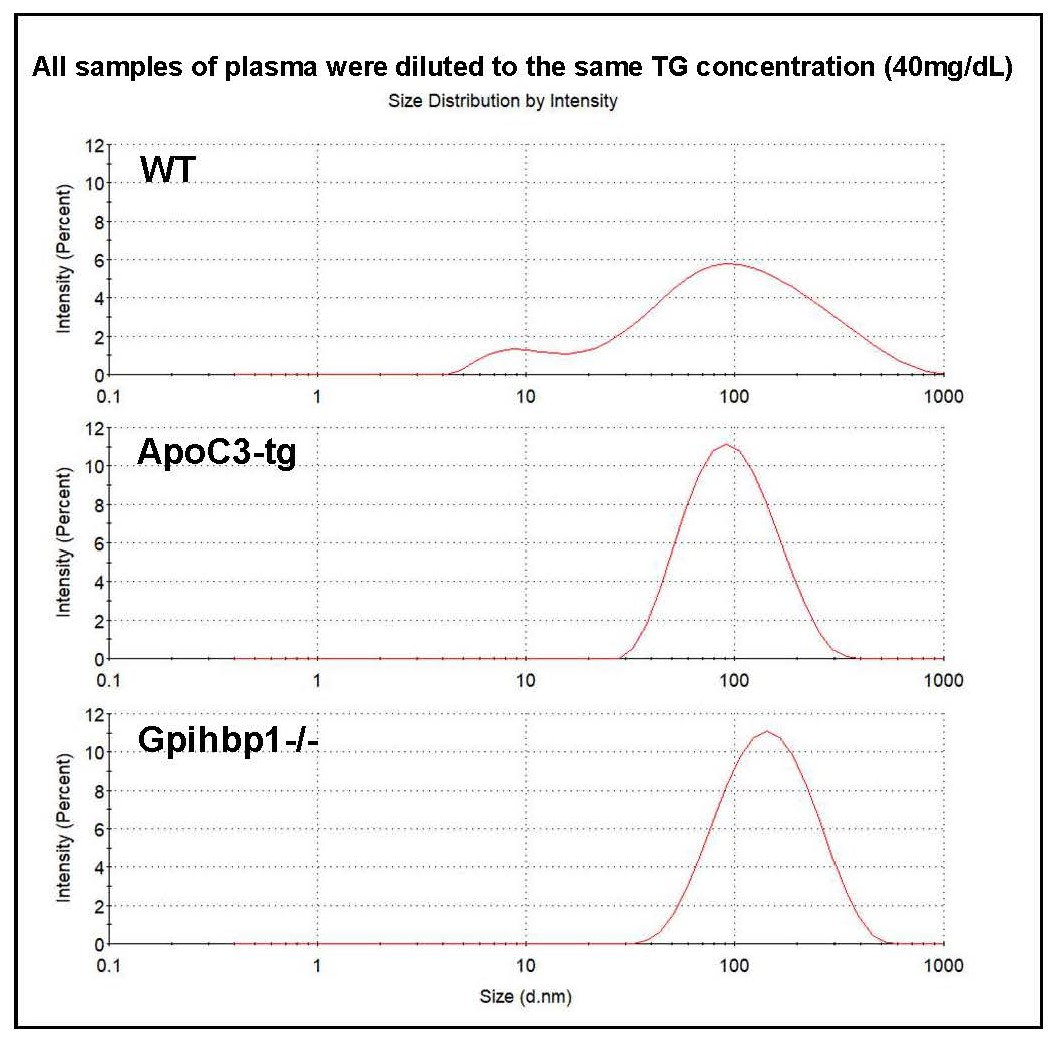


**Suppl Fig. 1** The representative graphs of particles distribution of plasma from wild-type, ApoC3-tg and Gpihbp1-/- mice. All plasma samples were diluted by normal saline to a triglyceride concentration of 40 mg/dL before measured by dynamic light scattering using a Malvern Zetasizer Nano ZS90. The plasma was collected from mice on a normal diet. WT, wild-type.





**Suppl Fig. 2** The range of plasma triglyceride levels of ApoC3-tg and Gpihbp1-/- mice, n=47 per group.


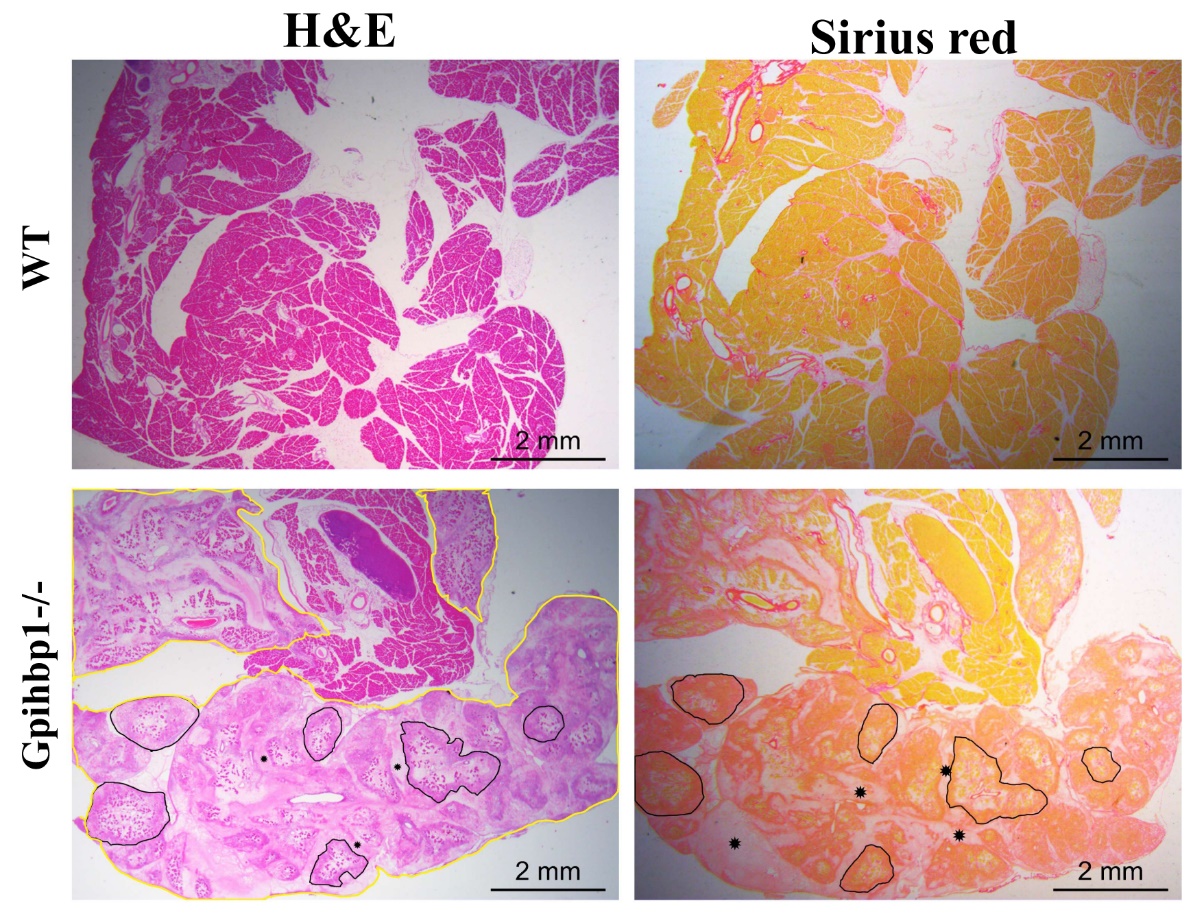


**Suppl Fig. 3** Illustration of experimental pancreatic necrosis in Gpihbp1-/- mice with caerulein-induced acute pancreatitis. Comparison of pathological features between necrotic tissue in HTG2 and non-necrotic tissue in WT mice. Necrotic tissues were distributed regionally and defined in patchy areas (outlined with yellow line) in H&E staining image (left column) in Gpihbp1-/- mice. There was a large amount of collagen fiber around the margin of lobule (outlined with black line) and collagen protein structure in the lobular septa (black star) in H&E staining (left column) and sirius staining images (right column).


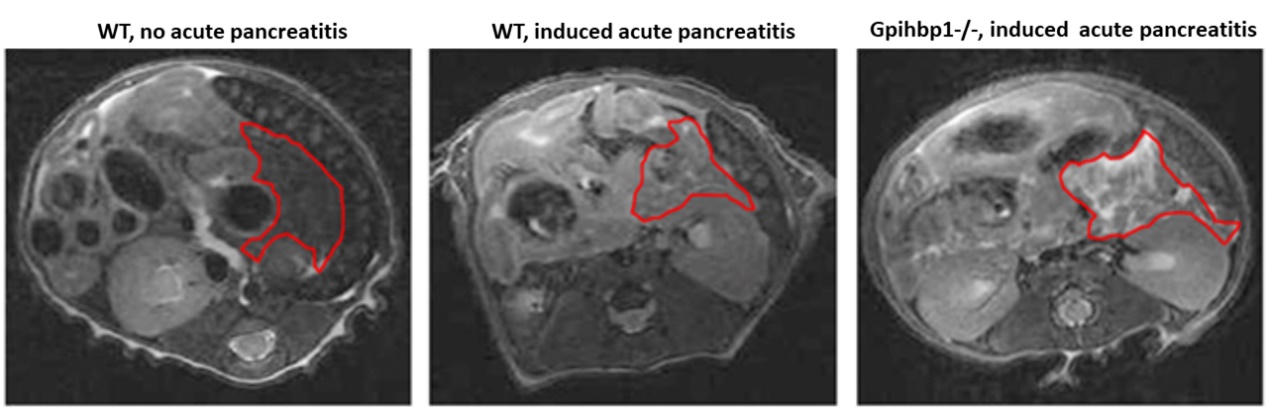


**Suppl Fig. 4** Features of the pancreas in magnetic resonance image in wild-type mouse injected with normal saline, as well as wild-type and Gpihbp1-/- mice in caerulein-induced acute pancreatitis. Red lines draw the outline of pancreas.


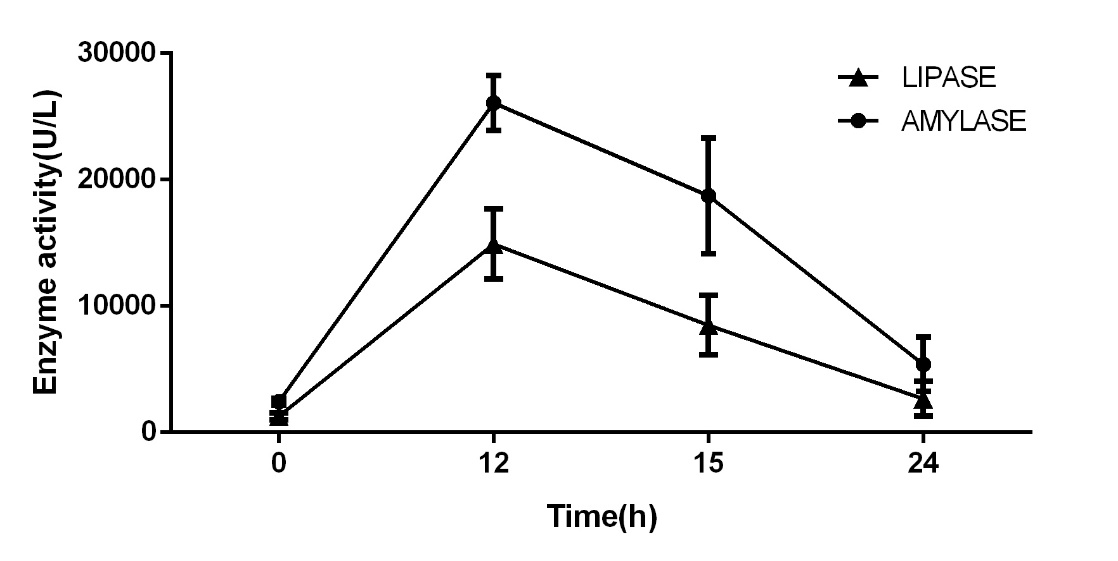


**Suppl Fig. 5** Plasma amylase and lipase activities of wild-type mice in various time point after the first injection of caerulein; Both plasma amylase and lipase activities reached to the peak at 12h after the first injection of caerulein (n=4 per group).





**Suppl Fig. 6** The plasma triglyceride levels of ApoC3-tg and Gpihbp1-/- mice before (0h) and after (12h) caerulein-induced acute pancreatitis. ** p<0.01; *** p<0.001; n=7-10 per group. Data was expressed as means ± SD in histograms. NS, no significant difference; TG, triglyceride; h, hour.


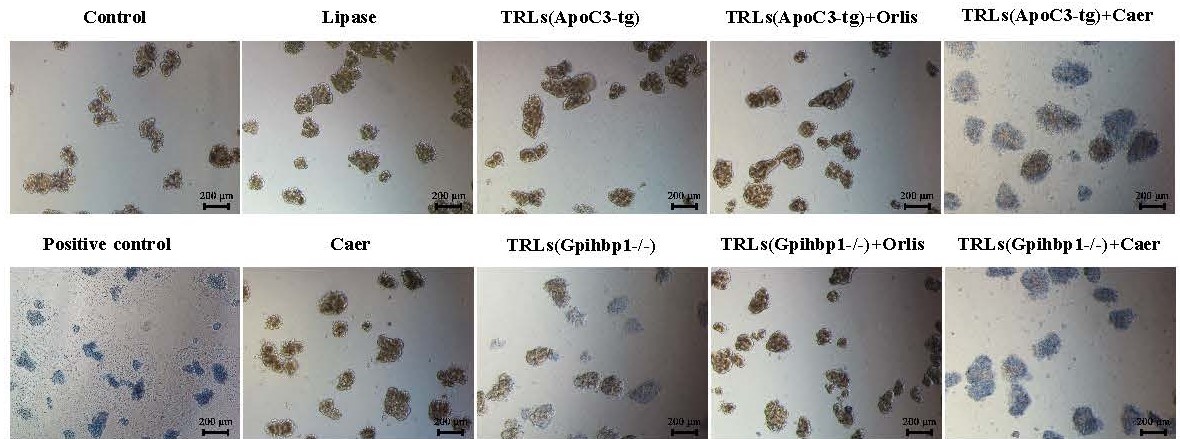


**Suppl Fig. 7** Representative graphs of Trypan Blue Dye of primary pancreatic acinar cells when incubated with caerulein, lipase, TRLs of ApoC3-tg and Gpihbp1-/- mice (triglyceride concentration, 40 mg/dL) alone or accompanied with caerulein or orlistat. Caer, Caerulein; Orlis, Orlistat.


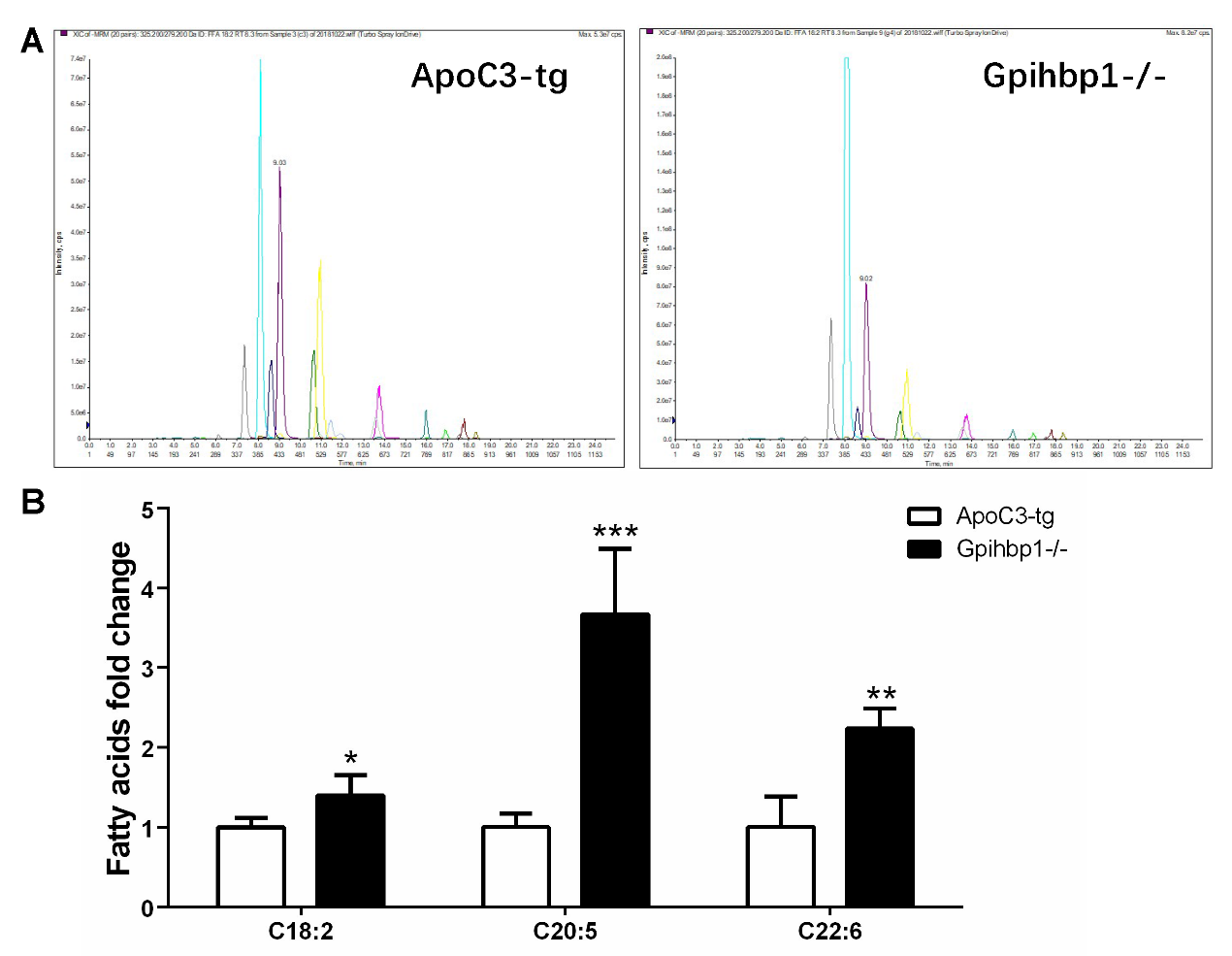


**Suppl Fig. 8** Fatty acid fraction analysis by LC-MS. (A) Representative graph of MS spectrum of the fatty acid fraction of the TRLs from ApoC3-tg and Gpihbp1-/- mice. (B) FFAs concentrations in TRLs from ApoC3-tg and Gpihbp1-/- mice before lipid extraction for further the fatty acid fraction analysis. (C) The relative difference of various types of FFA of TRLs between ApoC3-tg and Gpihbp1-/- mice. *p<0.05 or **p<0.01 or ***p<0.001 (n=4 per group). Data was expressed as means ± SD in histograms.


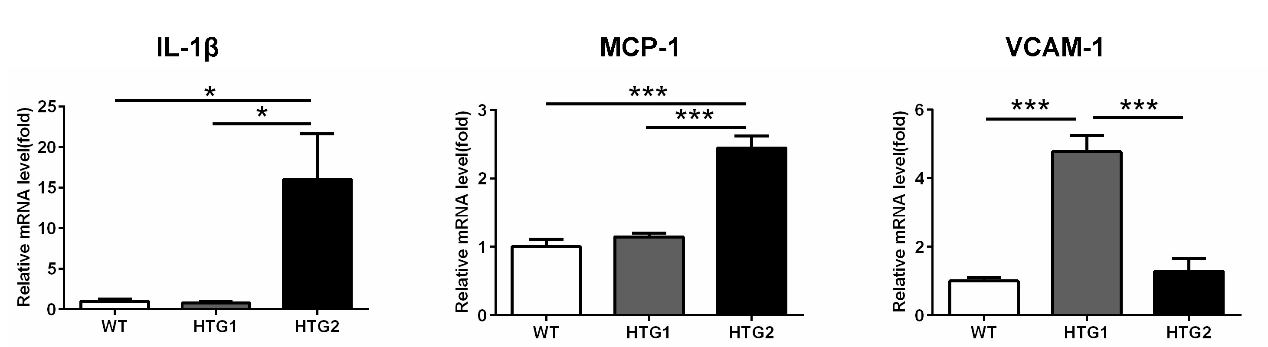


**Suppl Fig. 9** Relative mRNA levels of IL-1β, MCP-1 and VCAM-1 among WT, HTG1 and HTG2 mice in caerulein-induced acute pancreatitis. *p<0.05 or **p<0.01 or ***p<0.001 (n=3-6 per group). Data was expressed as means ± SD in histograms.
